# Sellar Suprasellar Surprise: A Rare Case of Atypical Teratoid/Rhabdoid Tumor in an Infant

**DOI:** 10.7759/cureus.47632

**Published:** 2023-10-25

**Authors:** Ravi Shah, Nivetha Ambalavanan, Debajyoti Chatterjee, Pinaki Dutta

**Affiliations:** 1 Endocrinology, Postgraduate Institute of Medical Education and Research, Chandigarh, IND; 2 Histopathology, Postgraduate Institute of Medical Education and Research, Chandigarh, IND; 3 Pathology, Postgraduate Institute of Medical Education and Research, Chandigarh, IND

**Keywords:** infant, vasopressin deficiency, hypopituitarism, ini1, integrase interactor 1, atypical teratoid/rhabdoid tumor

## Abstract

We present a case of a 10-month-old male infant who initially presented with polyuria, polydipsia, drowsiness, and fever. Neuroimaging using non-contrast computed tomography (NCCT) demonstrated obstructive hydrocephalus associated with a suprasellar mass, for which emergency neurosurgical intervention was performed with right parietal medium pressure ventriculoperitoneal (MPVP) shunting. For fever, no cause was found with sterile cerebrospinal fluid (CSF) analysis, and empirical antibiotics were administered. The patient exhibited polyuria with hypernatremia and was diagnosed with arginine vasopressin (AVP) deficiency, further complicated by visual impairment due to left optic atrophy. Hormonal workup revealed secondary hypothyroidism and hypocortisolism. Imaging by contrast-enhanced magnetic resonance imaging (CEMR) revealed a lobulated solid-cystic suprasellar mass with flow void, suggestive of adamantinomatous craniopharyngioma initially.

However, despite multiple neurosurgical interventions, the patient's condition deteriorated with recurrent fever and seizures, leading to a revision of ventriculoperitoneal shunts. Repeat CEMR showed an increase in the size of the lesion with spinal leptomeningeal metastasis, suggesting a different pathology. Transventricular biopsy confirmed an atypical teratoid and rhabdoid tumor (AT/RT), World Health Organization Classification of Tumors of the Central Nervous System (CNS WHO) grade 4, characterized by diffuse growth pattern, moderate nuclear pleomorphism, clear cytoplasm, and prominent nucleoli. Immunohistochemistry revealed positive vimentin staining and loss of integrase interactor 1 (INI1) expression, consistent with AT/RT.

The patient's parents were counseled on the need for multimodal management, including surgery and chemotherapy. However, due to socioeconomic constraints and a guarded prognosis, they chose to leave against medical advice. This case illustrates the diagnostic challenges in distinguishing AT/RT from other suprasellar masses and emphasizes the importance of a multidisciplinary approach in managing complex pediatric cases.

## Introduction

Sellar and suprasellar tumors constitute about 10% of all pediatric central nervous system (CNS) tumors. They encompass a wide range of distinct entities, having characteristic radiographic findings and histological origin [1]. One of the rarer tumors of the sellar suprasellar area is an atypical teratoid/rhabdoid tumor (AT/RT). AT/RT is a rare, aggressive embryonal tumor that comprises 1%-2% of pediatric CNS tumors but 10% of CNS tumors in infants [2].

AT/RT is characterized by reduced expression of the *SMARCB1* tumor suppressor gene, which encodes for SWI/SNF-related matrix-associated actin-dependent regulator of chromatin subfamily B member 1. It is present on chromosome 22q. It is responsible for coding of integrase interactor 1 (INI1) subunit of the SWI/SNF chromatin remodeling complex, which is involved in DNA double-strand break repair and nucleotide excision repair (NER) [3]. A review of 100 infants with AT/RT from the European Rhabdoid Registry (EU-RHAB) revealed that the cerebellum was the most common location of tumor, and sellar involvement was not seen [4]. A systematic review of 38 patients with sellar AT/RT revealed a median age of 44 years (range: 20-70 years) with a substantial female predominance (94.7%) [5]. In the above systematic review, no case was identified in infancy.

Here, we report a sellar suprasellar mass in a 10-month-old male, which turned out to be an AT/RT.

## Case presentation

**Figure 1:**
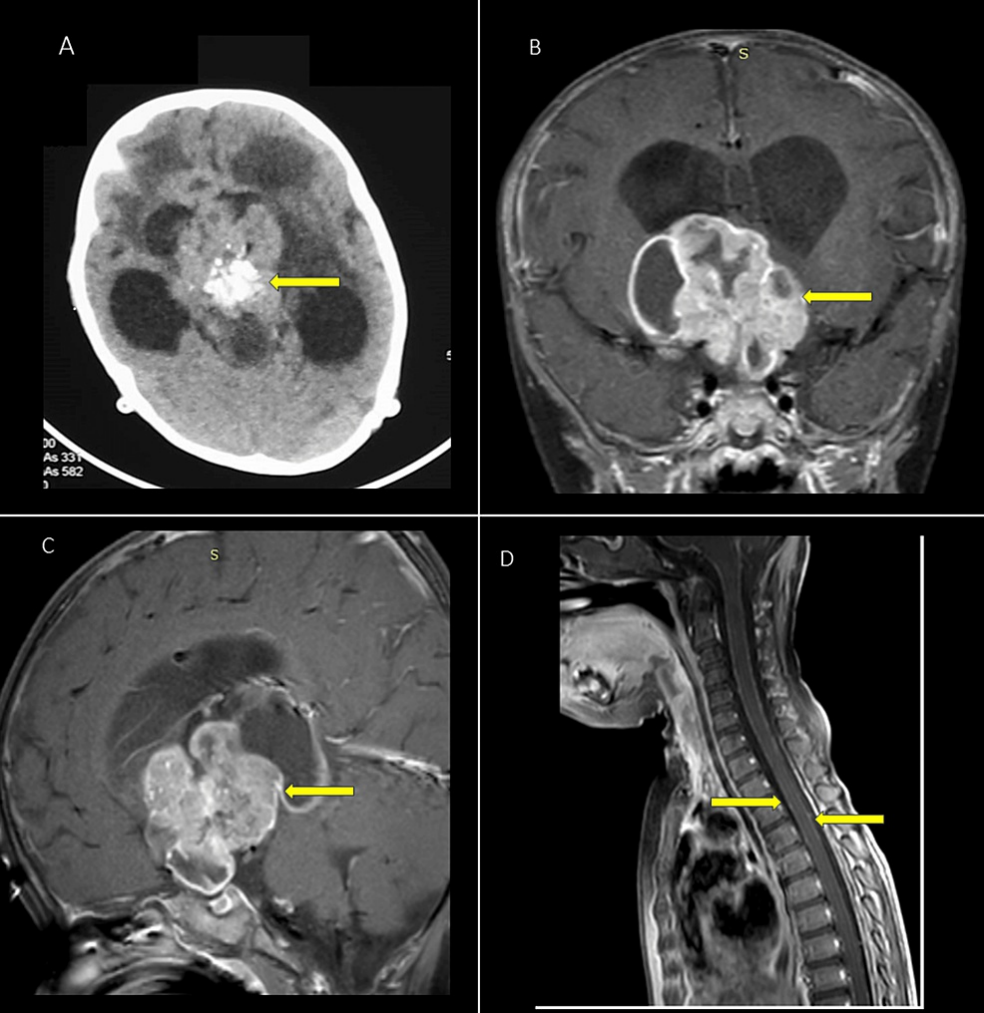
Radiological imaging of the sellar suprasellar mass and hydrocephalus in the presented case (A) Non-contrast CT scan showing the initial presentation of a sellar suprasellar mass (yellow arrow) with obstructive hydrocephalus relieved by MPVP shunt placement. Notably, calcification is observed within the lesion. (B and C) Contrast-enhanced T1-weighted MRI of the sellar and suprasellar area (coronal (B) and sagittal (C)) illustrating the solid-cystic nature of the sellar suprasellar mass with hydrocephalus and heterogeneous contrast enhancement (yellow arrow). (D) Contrast-enhanced T1-weighted MRI of the spine revealing leptomeningeal enhancement indicative of metastasis (yellow arrow). CT: computed tomography, MPVP: medium pressure ventriculoperitoneal, MRI: magnetic resonance imaging

A 10-month-old male, born out of a non-consanguineous marriage with a normal birth history, a birth weight of 3.5 kg, and normal milestones until six months of age, presented with complaints of polyuria, polydipsia, drowsiness, and fever for 15 days. Parents also noticed decreased crawling and feeding efforts. On examination, he had generalized hypotonia. Non-contrast computed tomography (NCCT) scan of the head was done, which was suggestive of obstructive hydrocephalus with a suprasellar mass. Emergency neurosurgery consultation was sought, and right parietal medium pressure ventriculoperitoneal (MPVP) shunting was done (Figure 1A).

For fever, no cause was found with sterile cerebrospinal fluid (CSF) analysis, so the patient was treated with empirical antibiotics. The patient was also analyzed for polyuria and found to have an output of 6 mL/kg/hour with serum sodium of 160 mEq/L with high serum osmolality(334 mOsm/kg) and low urine osmolality(94 mOsm/kg). He was given vasopressin 1 unit/m^2^ with a subsequent decrease in urine output and increase in urine osmolality suggestive of arginine vasopressin (AVP) deficiency. The patient had poor eye contact, and his response to confrontational stimuli was poor. The ophthalmological evaluation was suggestive of left optic atrophy with almost complete loss of vision on the right side. Repeat CSF analysis was done given a history of fever, which was normal. CSF alpha-fetoprotein (0.67 ng/mL) and human chorionic gonadotropin (HCG) (0.6 mIU/mL) were not found to be elevated. Family history was not significant. Hormonal workup was suggestive of secondary hypothyroidism and hypocortisolism, and the patient was supplemented for the same (Table 1).

**Table 1:** Biochemical and hormonal profile at presentation ACTH: adrenocorticotropic hormone, TSH: thyroid-stimulating hormone

Parameter	Patient value	Reference range
Hemoglobin (g/dL)	9	>12
Total leucocyte count (cells/μL)	4,500	4,000-11,000
C-reactive protein (mg/L)	1.1	<5
Procalcitonin (ng/mL)	0.02	<0.1
Creatinine (mg/dL)	0.82	0.5-1.2
Alanine transaminase/aspartate aminotransferase (U/L)	42/40	2-41/2-40
Serum sodium (mEq/L)	160	135-145
Serum osmolality baseline (mOsm/kg)	334	285-295
Urine osmolality baseline (mOsm/kg)	94	50-1,200
Urine osmolality post-vasopressin (mOsm/kg)	534	50-1,200
Basal growth hormone (ng/mL)	0.536	0-2.5
Insulin-like growth factor-1 (ng/mL)	7	18-116
Prolactin (ng/mL)	74.6	4.79-23.3
Total T4 (mcg/dL)	2.91	4.8-12.7
Cortisol (mcg/dL)	1.145	6.19-19.57
ACTH (pg/mL)	8	7.2-63.3
TSH (mcIU/mL)	1.21	0.27-4.2

Contrast-enhanced magnetic resonance imaging (CEMR) of the hypothalamus and pituitary area was done, which revealed a 4.3 × 4.6 × 3.8 cm lobulated, solid-cystic suprasellar mass with flow void, which was corroborated with CT and found to be internal foci of calcification. It was hyperintense on T2-weighted imaging and iso-hypointense on T1-weighted imaging with heterogeneous post-contrast enhancement (Figure 1D, 1E). Given the age of presentation and solid-cystic nature, the initial possibility of adamantinomatous craniopharyngioma was kept.

Post-MPVP shunting, the patient again had a recurrence of fever and episodes of seizures with no improvement in symptoms. Hence, antiepileptics were stepped up, and repeat NCCT was done, which was suggestive of gross hydrocephalus with impaired function of the shunt. The patient underwent revision of the right shunt, followed by placement of another shunt on the left side as well. Post-procedure, there was a decrease in seizure frequency, but polyuria persisted, and the patient was treated with vasopressin infusion. Repeat CEMR sella was suggestive of an increase in the size of the lesion to 4.7 × 3.5 × 5.9 cm (30% increase in tumor volume as compared to baseline within one month) with spinal leptomeningeal metastasis (Figure 1C), and given temporal progression, it made craniopharyngioma less likely. Ultrasound of the abdomen, done to look for renal or abdominal mass, was normal. The patient underwent a transventricular biopsy of the suprasellar mass with repeat CT post-procedure, showing no complication post-procedure (Figure 1B).

**Figure 2:**
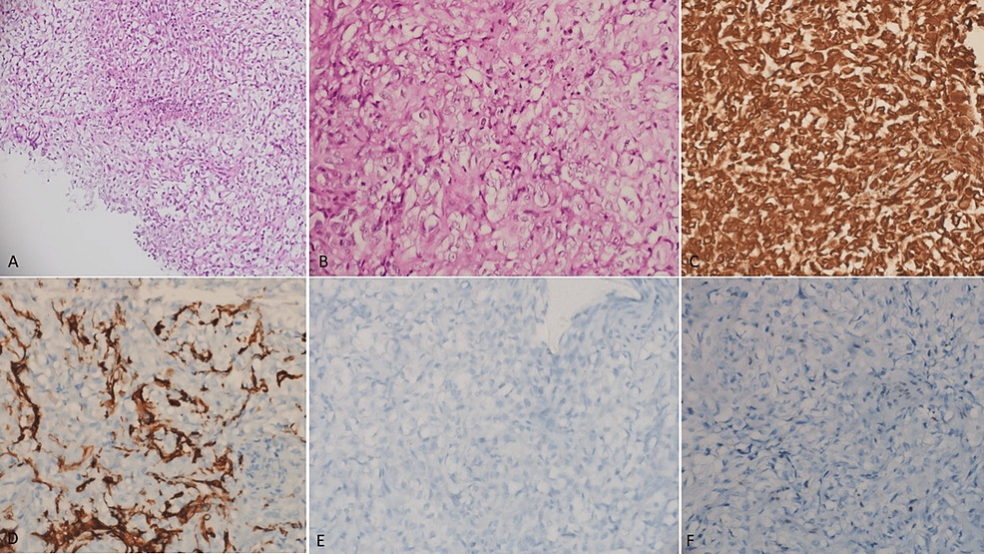
Histopathological and immunohistochemical evaluation of the suprasellar tumor (A) Biopsy shows a cellular tumor arranged in a diffuse pattern and nests separated by delicate thin-walled blood vessels (hematoxylin and eosin, ×200). (B) The tumor cells exhibit clear cytoplasm and a round nucleus with a hyperchromatic nucleus with a few mitotic activities (hematoxylin and eosin, ×400). (C) Immunohistochemical staining demonstrating diffuse positivity for vimentin, indicating mesenchymal differentiation (magnification, ×400). (D) Focal patchy positivity for GFAP within the tumor cells (magnification, ×400). (E) Negative staining for OCT3/OCT4, ruling out germinoma (magnification, ×400). (F) Loss of expression of INI1 in tumor cells (retained expression in endothelial cells) (magnification, ×400), which is characteristic of AT/RT. These histopathological and immunohistochemical findings supported the diagnosis of AT/RT, CNS WHO grade 4, in this complex pediatric case. GFAP: glial fibrillary acidic protein, OCT3/OCT4: octamer-binding transcription factor 3 and 4, INI1: integrase interactor 1, AT/RT: atypical teratoid/rhabdoid tumor, CNS WHO: World Health Organization Classification of Tumors of the Central Nervous System

The histopathology showed a tumor arranged predominantly in a diffuse pattern with few nests separated by thin fibrovascular septa with individual tumor cells showing moderate nuclear pleomorphism, with moderate to abundant clear cytoplasm, and a prominent nucleolus (Figure 2A, 2B). Brisk mitotic activity was noted. Based on histology, possibilities of germ cell tumor, aggressive pituitary neuroendocrine tumor, and AT/RT were considered. On immunohistochemistry, the tumor cells showed diffuse positivity for vimentin (Figure 2C) and focal patchy positivity for pancytokeratin and glial fibrillary acidic protein (GFAP) (Figure 2D). The tumor cells showed loss of expression of integrase interactor (INI) (retained expression in endothelial cells) (Figure 2F) and were negative for synaptophysin and chromogranin (ruling out neuroectodermal tumors and glioma). Octamer binding transcription factor 3 and 4 (OCT3/OCT4) was negative (ruling out germinoma) (Figure 2E), with thyroid transcription factor 1 (TTF1) negative in tumor cells (ruling out posterior pituitary tumors) (Figure 2D). This histopathological and immunohistochemical profile was consistent with atypical teratoid and rhabdoid tumor (World Health Organization Classification of Tumors of the Central Nervous System (CNS WHO) grade 4).

The patient's parents were explained regarding the need for multimodality management including surgery and chemotherapy. However, they refused further treatment because of socioeconomic constraints and guarded prognosis and left against medical advice. Till the report of the case, the patient was in stable condition.

## Discussion

The differential diagnosis for sellar suprasellar tumors in infancy is quite vast as shown in Table 2. Our index case presented with sellar suprasellar mass and was initially suspected to have adamantinomatous craniopharyngioma in view of solid-cystic mass with calcification, age of presentation, and associated AVP deficiency. However, repeat imaging was suggestive of rapid enlargement of the tumor with seeding of the neuraxis, and histopathological examination revealed an AT/RT.

**Table 2:** Differential diagnosis of sellar suprasellar tumors in infancy AT/RT: atypical teratoid/rhabdoid tumor, MEN1: multiple endocrine neoplasia type 1, NF1: neurofibromatosis type 1

Various sellar suprasellar tumors in infancy
Pilocytic astrocytoma, gliomas, glioneuronal and neuronal tumors
Embryonal tumors including AT/RT
Histiocytic tumors and other hematolymphoid tumors
Germ cell tumors
Sellar tumors: adamantinomatous and papillary craniopharyngioma, PitNET, pituicytoma and other posterior pituitary tumors, pituitary blastoma
Genetic tumor syndromes: MEN1, NF1, and DICER1 syndrome

Clinically and radiologically, it is challenging to differentiate AT/RT of the sellar suprasellar area from other tumors. Clinically, they present with headache, diplopia, and features of arginine vasopressin deficiency and secondary pituitary hormonal deficiencies. On imaging, they are characterized by tumors with solid, cystic, and necrotic areas. On CT, calcification is present in 50%, which was seen in our patient. On MRI, signal intensity is variable in T1- and T2-weighted imaging; however, diffusion restriction and post-contrast enhancement are typical [2]. To differentiate AT/RT from other tumors of the sellar suprasellar region, two characteristics are particularly helpful. AT/RT are aggressive; hence, they are characterized by rapid enlargement in the size of the tumor on follow-up imaging. Also, AT/RT has a propensity for seeding the neuraxis, and distant metastasis is seen in one-third of cases [6]. The index case had a 30% enlargement in tumor volume with seeding to spinal leptomeninges in imaging done one month apart, hence fulfilling both characteristics. Normal CSF alpha-fetoprotein and beta-HCG further re-emphasize other possibilities, making the possibility of germ cell tumors remote. The immunohistochemical hallmark of AT/RT is loss of INI1 expression as seen in the index case [7].

There is no consensus on the treatment of AT/RT; however, most patients are given multimodality treatment including surgery, radiotherapy, chemotherapy, and autologous hematopoietic stem cell transplantation. However, radiotherapy is generally not offered to infants, and only surgery and chemotherapy are offered. They are associated with poor prognosis despite the above, especially when they originate at age <3 years [8]. The chemotherapeutic regimen of choice is not well defined; however, the commonly used options include ifosfamide, carboplatin, Adriamycin, temozolomide, and etoposide [9]. Patients who received combined chemoradiation therapy had a significantly increased overall survival (25 months higher) compared to those who received single modality or no adjuvant therapies in a systematic review of patients with sellar atypical rhabdoid teratoid tumor [5].

## Conclusions

In conclusion, we report a rare and challenging case of a 10-month-old infant presenting with a suprasellar mass, hydrocephalus, and a constellation of endocrine abnormalities. Initially, the clinical presentation mimicked adamantinomatous craniopharyngioma, a common pediatric suprasellar tumor. However, the subsequent clinical course, including recurrent fever, seizures, and rapid tumor progression with leptomeningeal metastasis, led to the diagnosis of an atypical teratoid and rhabdoid tumor (AT/RT), a highly aggressive malignancy. This case underscores the importance of considering rare neoplastic entities, such as AT/RT, in the differential diagnosis of suprasellar masses, particularly when clinical features and radiological findings do not align with the expected course of more common tumors. Furthermore, the coexistence of endocrine dysfunction, including AVP deficiency, secondary hypothyroidism, and hypocortisolism, highlights the intricate interplay between tumor location and hormonal regulation in the hypothalamic-pituitary axis.

Early recognition and prompt multidisciplinary management are crucial in cases of pediatric suprasellar tumors, especially when associated with hydrocephalus and endocrine abnormalities. While AT/RT remains a formidable diagnostic and therapeutic challenge, accurate and timely diagnosis is essential for optimizing treatment strategies and improving patient outcomes. This case serves as a reminder of the need for a comprehensive and individualized approach to managing complex pediatric endocrine and neurosurgical conditions.
